# Cryptotanshinone Regulates Androgen Synthesis through the ERK/c-Fos/CYP17 Pathway in Porcine Granulosa Cells

**DOI:** 10.1155/2017/5985703

**Published:** 2017-01-12

**Authors:** Danfeng Ye, Meifang Li, Yuehui Zhang, Xinhua Wang, Hua Liu, Wanting Wu, Wanying Ma, Kewei Quan, Ernest H. Y. Ng, Xiaoke Wu, Maohua Lai, Hongxia Ma

**Affiliations:** ^1^Guangzhou Medical University, Guangzhou 510120, China; ^2^Department of Obstetrics and Gynecology, First Affiliated Hospital, Heilongjiang University of Chinese Medicine, Harbin 150040, China; ^3^Center for Post-Doctoral Studies, Heilongjiang University of Chinese Medicine, Harbin 150040, China; ^4^Research Institute of Integrated Traditional Chinese Medicine and Western Medicine, Guangzhou Medical University, Guangzhou 510120, China; ^5^Department of Traditional Chinese Medicine, The First Affiliated Hospital of Guangzhou Medical University, Guangzhou 510120, China; ^6^Department of Obstetrics and Gynecology, Queen Mary Hospital, University of Hong Kong, Pok Fu Lam 999077, Hong Kong

## Abstract

The aim of the study is to investigate the molecular mechanism behind androgen reduction in porcine granulosa cells (pGCs) with* Salvia miltiorrhiza* Bunge extract cryptotanshinone. PGCs were isolated from porcine ovaries and identified. Androgen excess model of the pGCs was induced with the MAPK inhibitor PD98059 and then treated with cryptotanshinone. The testosterone level was measured by radioimmunoassay in the culture media. The protein levels of P-ERK1/2, c-Fos, and CYP17 in the cells were measured by western blot. Cryptotanshinone decreased the concentration of testosterone and the protein level of CYP17 and increased the protein levels of P-ERK1/2 and c-Fos in the androgen excess mode. After the c-Fos gene was silenced by infection with c-Fos shRNA lentivirus, we measured the mRNA expression by quantitative RT-PCR and protein level by western blot of P-ERK1/2, c-Fos, and CYP17. This showed that the mRNA expression and protein level of P-ERK1/2 and c-Fos were significantly reduced in the shRNA–c-Fos group compared to the scrambled group, while those of CYP17 were significantly increased. So we concluded that cryptotanshinone can significantly reduce the androgen excess induced by PD98059 in pGCs. The possible molecular mechanism for this activity is regulating the ERK/c-Fos/CYP17 pathway.

## 1. Introduction

Polycystic ovary syndrome (PCOS) is a common endocrine disorder [1] associated with anovulation/oligoovulation, hyperandrogenism, and polycystic ovaries [2]. It is also associated with increased risk of cardiovascular diseases [3, 4] and type 2 diabetes [5]. PCOS is believed to affect approximately 10% of all women of reproductive age worldwide [6, 7], but the pathophysiology of the ovulatory dysfunction in PCOS remains poorly understood. Hyperandrogenism is considered to be one of the most important factors [8], and increased androgen concentration in the follicular fluid is associated with elevated serum levels of luteinizing hormone that can arrest follicle development and lead to follicular degeneration [9]. Elevated testosterone, either directly or indirectly, has been shown to decrease the success rate of in vitro maturation, fertilization, and embryonic development [10]. In addition, high testosterone concentration is associated with a higher risk of miscarriage in women with PCOS [11]. Therefore, reducing androgen levels is critical to treating PCOS.

Because androgen synthesis is such a complicated process, there are many different processes that can be targeted for reducing androgen levels. At present, the most commonly used antiandrogen drugs include oral contraceptives, androgen receptor antagonists, and 5*α*-reductase inhibitors. However, adverse reactions to these drugs have been reported. For example, long-term use of oral contraceptives is associated with increased risks of thromboembolism [12] and cardiovascular diseases [13], and spironolactone (an androgen receptor antagonist) can cause irregular menstruation, hyperkalemia, and hypotension. Thus it is necessary to find safer and more effective methods to control the androgen excess associated with PCOS.

Traditional Chinese drugs, which are extracted from natural medicinal herbs, are believed to be much safer than synthetic drugs [14]. These traditional drugs have been widely used for the treatment of hyperandrogenism in China and other Asian countries for many years, and their efficacy has recently been affirmed [15–17]. An important aspect of these drugs is that they have multiple targets. Cryptotanshinone, traditionally known as tanshinone, was originally isolated from the dried roots of* Salvia miltiorrhiza* Bunge [18] and is commonly used to treat acne due to its ability to reduce testosterone levels [19]. Studies have shown that cryptotanshinone can reduce the levels of 17-OHP, the precursor of testosterone, in prenatally androgenized male rats [20] and can reverse reproductive and metabolic disturbances in prenatally androgenized female rats via regulation of CYP17 levels and other molecules in the insulin signaling pathway [21]. One study suggested that cryptotanshinone can reduce hyperandrogenism in PCOS women by decreasing the synthesis of steroidal hormones in the theca cells [22]. Although these studies showed that cryptotanshinone could reduce androgen synthesis, they did not establish the molecular mechanism behind this effect. The primary goal of the present study, therefore, was to determine the molecular mechanism for androgen reduction by cryptotanshinone.

Blocking extracellular signal regulated kinase (ERK) pathway by inhibitor PD98059 has been shown to induce androgen excess [23, 24]. We cultured porcine granulosa cells (pGCs) with PD98059 to induce the androgen excess phenotype and then treated the cells with cryptotanshinone to determine if it could reduce androgen excess in these cells and, if so, by what mechanism. Our results suggest that cryptotanshinone might contribute to the development of novel therapies for treating androgen excess in PCOS patients.

## 2. Materials and Methods

### 2.1. In Vitro Cell Culture

Porcine ovaries were obtained from JiaHe Slaughterhouse in Guangzhou. Ovaries were removed and immediately placed into warm 0.9% sodium chloride solution containing 1% penicillin and 1% streptomycin (Sigma, St Louis, MO, USA) and transported to the laboratory within one hour. Ovaries were washed with phosphate buffered saline (PBS) three times. The pGCs were aspirated from follicles 1 mm to 5 mm in diameter and placed into a sterile centrifuge tube containing 0.9% sodium chloride solution and centrifuged at 1500 ×g for 5 minutes. The supernatant was removed and the cells were washed once with DMEM (HyClone, Logan City, Utah, USA) for three times. The pGCs were dispersed by repeated pipetting and suspended in fresh complete medium (DMEM comprising 10% FBS and 1% penicillin and streptomycin). The cell suspension was collected and seeded in 6-well plates at a density of 1 × 10^6^ cells/well and cells were incubated in a humidified incubator with 95% air and 5% CO_2_ at 37°C for 24 hours.

### 2.2. Induction of Androgen Excess in pGCs by PD98059

After culturing for 24 hours, the medium was discarded and the pGCs were washed with DMEM. The pGCs were then treated with PD98059 or with complete medium as a control. To determine the best dose of PD98059 (Selleckchem, Houston, USA) and the best cell-culture time to induce androgen excess in pGCs, we performed the following two tests. First, PD98059 was added to the cell cultures at 0 *μ*M, 1 *μ*M, 3 *μ*M, 10 *μ*M, and 25 *μ*M and the culture time was 24 h. The culture media were collected to measure the testosterone levels, and the cells were collected to run western blots. The highest level of androgen production was with 10 *μ*M PD98059 (Figures 2(a) and 3(a)). The second test used cell cultures of 0 h, 1 h, 5 h, 24 h, 48 h, and 72 h in the presence of 10 *μ*M PD98059, and we found out that the testosterone level and the protein level of CYP17 were maximized at 24 hours (Figures 2(b) and 3(b)).

### 2.3. Intervention with Cryptotanshinone in the Androgen Excess Cell Model

To investigate the efficacy of reducing androgen excess with cryptotanshinone, we performed the following experiments. After inducing the androgen excess cell model as described above, cell-culture medium containing PD98059 was removed. Then we further cultured the androgen-producing cells with cryptotanshinone (Sigma, Canada) at 0 *μ*M, 10 *μ*M, 25 *μ*M, and 50 *μ*M for another 24 h. We found that 10 *μ*M was the best concentration for reducing androgen levels. We then cultured the cells with 10 *μ*M cryptotanshinone for 3 h, 6 h, 18 h, and 24 h, and testosterone levels and protein levels were determined as described below.

### 2.4. Infection with c-Fos shRNA Lentivirus

The pGMLV-SC1-shRNA plasmid carrying either a scrambled or c-Fos short hairpin RNA sequence was 5′–gatcc GGA GAC AGA CCA GCT AGA AGA TTC AAG AGA TCT TCT AGC TGG TCT GTC TCC TTT TTT g–3′. Lentiviruses were packed using human embryonic kidney 293T cells. The viruses were harvested and the titer of which was tested and then used to infect the pGCs. Multiplicity of infection (MOI) was calculated based on the number of viable pGCs plated. The pGCs were plated in DMEM with antibiotics and FBS to permit cell anchorage. After a 24-hour attachment period, primary monolayer cultures of pGCs in 6-well plates were infected for 24 h in DMEM with 10% FBS and c-Fos shRNA lentivirus (MOI = 30). After a 24 h infection period, the medium was replaced with DMEM containing 1% antibiotics and 10% FBS and the culture was continued for another 48 h. After the incubation period, the cells were rinsed with PBS and protein and RNA were extracted for western blot analysis and quantitative RT-PCR, respectively.

### 2.5. Measurement of Androgen Levels

Testosterone concentrations in the culture medium were measured using a testosterone radioimmunoassay kit (Beijing North Institute of Biotechnology). The intra- and interassay variations were less than 10% and 15%, respectively, and the sensitivity was 0.02 ng/mL.

### 2.6. Western Blot

Whole-cell extracts were prepared in radioimmunoprecipitation assay buffer (50 mM Tris (pH 7.4), 150 mM NaCl, 1% NP-40, 0.1% SDS, 1.5 *μ*M EDTA, 2 *μ*g/mL leupeptin, 2 *μ*g/mL Aprotinin, and 1 mM NaVanadate) with 1 *μ*M phenylmethanesulfonyl fluoride incubated for 30 min on ice and centrifuged for 15 min at 14000 ×g at 4°C. The supernatant was saved as a whole protein fraction. Protein concentrations were determined with a BCA protein assay kit. Using SDS-PAGE electrophoresis flat vertical separation gel concentrations of 10%, PH 8.8, concentrated gel concentration 4%, PH 6.8, and the electrode using Tris-glycine buffer, 100 V, the run time is 90 minutes. After SDS-PAGE, the protein (30 *μ*g) was transferred to nitrocellulose membranes. The membrane was blocked for 1 h at room temperature in Tris-buffered saline and 0.1 Tween containing 5% low-fat milk powder. The membranes were then incubated overnight at 4°C with one of the following primary antibodies: anti-P-ERK1/2 (ERK1/2 phospho-Thr202/Thr204) (1 : 1000 dilution, Cell Signaling Technology, Boston, MA), anti-c-Fos (1 : 400, Santa Cruz Biotechnology, Dallas, TX), anti-CYP17 (1 : 800, Biorbyt, UK), or anti-GAPDH (1 : 1000; Hangzhou XianZhi Biotech Company, China). The membranes were washed three times in TBST for 10 min and then incubated with horseradish peroxidase-tagged secondary antibodies (1 : 3000, Santa Cruz Biotechnology). Blots were developed with an enhanced chemiluminescence kit as described by the manufacturer (Amersham Pharmacia Biotech, Buckinghamshire, UK). Immunoreactivity was quantified by densitometric scanning with Quantity One software (Bio-Rad, San Diego, CA). Band intensities were normalized to GAPDH.

### 2.7. PCR and Quantitative RT-PCR

Cells were lysed in 1 mL of TRIzol Reagent (Invitrogen). cDNA was reverse transcribed from mRNA using PrimeScript RT Master Mix (TaKaRa).

1 uL cDNA was taken to perform PCR using Taq DNA polymerase (Qiagen) with *α* estrogen receptor primer and *β* estrogen receptor primer, respectively, under the same conditions. The conditions were 5 min at 95°C firstly and then 30 s at 95°C, 30 s at 55°C, and 30 s at 72°C for 35 cycles and 5 min at 72°C finally. The primer sequences were as follows: *α* estrogen receptor upstream primer 5′-CCCTACACACCAAAGCGTCC-3′ and downstream primer 5′-GCCCGACTGGCCGTAGAC-3′ and *β* estrogen receptor upstream primer 5′-ACACCTCTCTCCTTTAGCC-3′ and downstream primer 5′-CCTGACGCATAATCACTG-3′. All primers were synthesized using Shanghai Sangon Biological Technology (Guangzhou, China). The product lengths of *α* estrogen receptor primer and *β* estrogen receptor primer were 221-base pair (bp) and 239 bp. PCR products were electrophoresed in 1.5% agarose gels. After the electrophoresis, the gel was analysed with Image Lab (Bio-Rad).

Quantitative PCR (qPCR) was performed with SYBR premix EX Taq (TaKaRa). Total RNA was isolated from untreated, c-Fos shRNA-treated, and scrambled shRNA-treated pGCs. Equivalent dilutions of the resulting cDNA ware used to perform qPCR amplification of total ERK1/2 mRNA, c-Fos mRNA, and CYP17 mRNA. qPCR primers were designed using the primer designing tool (primer 3). The sequences of oligonucleotides were as follows: ERK1/2 upstream primer 5′-CAC TGG CTT TCT GAC CGA GT-3′ and downstream primer 5′-GTG ATG CGC TTG TTT GGG TT-3′; c-Fos upstream primer 5′-TCA GAG CAT TGG CAG AAG GG-3′ and downstream primer 5′-GTG AGC TGC CAG GAT GAA CT-3′; CYP17 upstream primer 5′-TGT CGT CGT CAA TCT GTG GG-3′ and downstream primer 5′-GGG TGG AGT CAG GAG GTA CT-3′; and *β*-actin upstream primer 5′-TCT ACA CCG CTA CCA GTT CG-3′ and downstream primer 5′-TCG ATG GGG TAC TTG AGG GT-3′ as an internal control. The qPCR cycle parameters for all primers were an initial denaturation for 30 s at 95°C, 40 cycles of 5 s at 95°C and 30 s at 60°C, and a final dissociation stage of 15 s at 95°C. Final results were expressed as the fold differences in gene expression relative to the normalized calibrator as calculated by 2^−ΔΔCt^ method [25].

### 2.8. Statistical Analysis

Values are expressed as mean ± SD. Differences among groups were tested by analysis of variance (ANOVA) with Bonferroni adjustments or by Student's* t*-test. A value of *p* < 0.05 was considered statistically significant. All statistical evaluations were performed with SPSS software (version 17.0, SPSS).

## 3. Results

### 3.1. Identification of the pGCs

**Figure 1:**
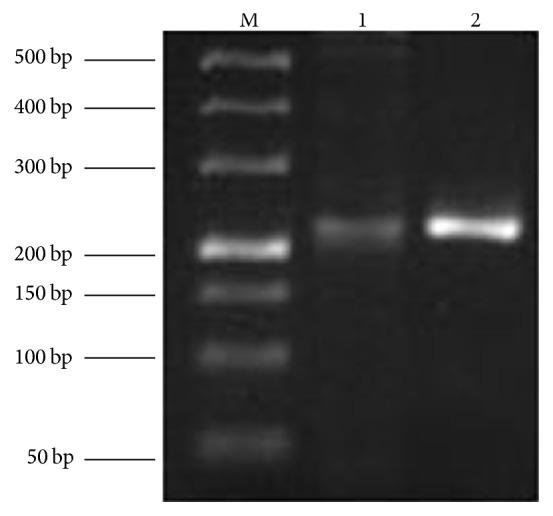
The detection of PCR for specific primers. M: 500 bp DNA marker. 1: *α* estrogen receptor gene; 2: *β* estrogen receptor gene.

The plot of *β* estrogen receptor, which is characteristically found in ovary granulosa cells differentiated from ovary theca cells that express *α* estrogen receptor gene [26, 27], was much brighter and deeper at 239 bp position than that of *α* estrogen receptor at 221 bp position (Figure 1), showing that most of the cells cultured in the study are granulosa cells.

### 3.2. The Phenotype of pGCs after Inhibiting the MAPK Pathway with PD98059

**Figure 2:**
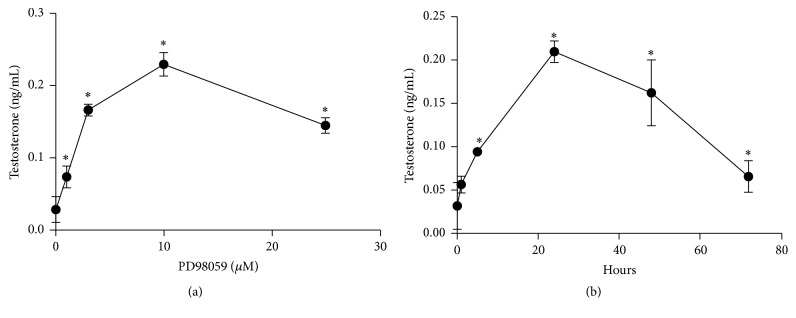
The androgen excess phenotype in porcine granulosa cells induced by PD98059 (values are mean ± SEM). (a) The testosterone level with 0 *μ*M, 1 *μ*M, 3 *μ*M, 10 *μ*M, and 25 *μ*M PD98059 for 24 h. ^*∗*^*p* < 0.05 versus 0 *μ*M (one-way ANOVA). (b) The testosterone level after culture times of 0 h, 1 h, 5 h, 24 h, 48 h, and 72 h with 10 *μ*M PD98059. ^*∗*^*p* < 0.05 versus 0 h (one-way ANOVA). All experiments were repeated at least three times.

**Figure 3:**
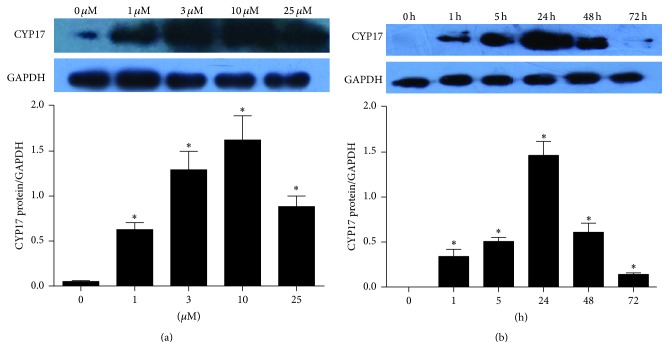
The protein expression of CYP17 in cells after exposure to PD98059 (values are mean ± SEM). (a) The protein level of CYP17 when PD98059 was 0 *μ*M, 1 *μ*M, 3 *μ*M, 10 *μ*M, and 25 *μ*M and the culture time was 24 h. ^*∗*^*p* < 0.05 versus 0 *μ*M (one-way ANOVA). (b) The protein expression of CYP17 when the culture time was 0 h, 1 h, 5 h, 24 h, 48 h, and 72 h with 10 *μ*M PD98059. ^*∗*^*p* < 0.05 versus 0 h (one-way ANOVA). All experiments were repeated at least three times.

The testosterone and CYP17 protein levels increased significantly when treated with PD98059 (*p* < 0.05) and were greatest at 10 *μ*M PD98059 (*p* < 0.01) (Figures 2(a) and 3(a)). In the presence of 10 *μ*M PD98059, the testosterone and CYP17 protein levels both increased significantly at all time points (*p* < 0.05) and were highest at 24 h (*p* < 0.01) (Figures 2(b) and 3(b)). Therefore, 10 *μ*M PD98059 for 24 h was chosen for inducing the androgen excess phenotype in pGCs in subsequent experiments.

### 3.3. The Effectiveness of Reducing Androgen Excess with Cryptotanshinone

**Figure 4:**
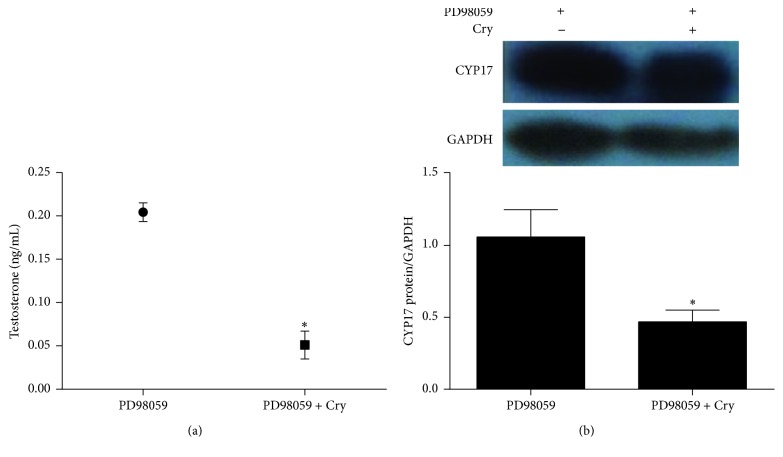
The effectiveness of reducing androgen excess in pGCs with cryptotanshinone (values are mean ± SEM). (a) The testosterone level in the medium of androgen excess cells treated with or without cryptotanshinone (10 *μ*M) for 6 h (^*∗*^*p* < 0.05, Student's* t*-test). (b) The protein expression of CYP17 in androgen excess pGCs treated with or without cryptotanshinone (10 *μ*M) for 6 h (^*∗*^*p* < 0.05, Student's* t*-test). All experiments were repeated at least three times.

**Figure 5:**
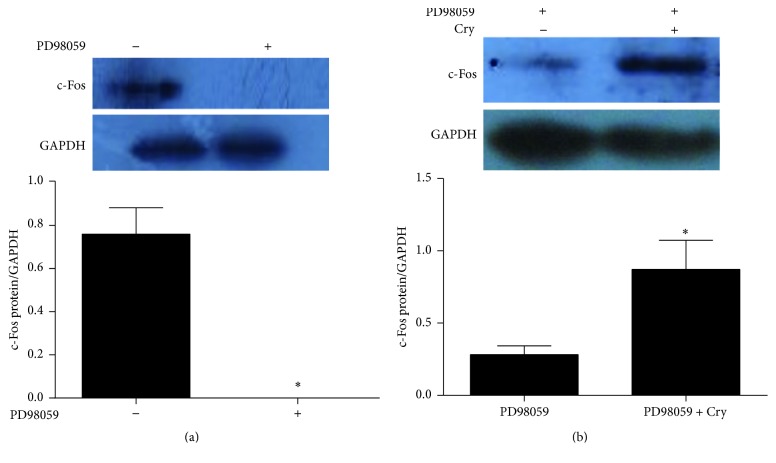
The effects of cryptotanshinone on c-Fos protein level in pGCs (values are mean ± SEM). (a) The protein level of c-Fos in pGCs when treated with PD98059 (10 *μ*M) for 24 h. ^*∗*^*p* < 0.01 versus no PD98059 (one-way ANOVA). (b) The protein level of c-Fos in androgen excess cells when treated with cryptotanshinone (10 *μ*M) for 6 h. ^*∗*^*p* < 0.05 versus complete medium (one-way ANOVA). All experiments were repeated at least three times.

Both the testosterone and CYP17 levels decreased significantly after treatment with 10 *μ*M cryptotanshinone for 6 h (*p* < 0.05Figure 4(a)). These results showed that cryptotanshinone can reverse the androgen excess in pGCs induced by PD98059. We also found that the c-Fos protein level was significantly decreased after exposure to PD98059 (*p* < 0.01Figure 5(a)) and that these levels returned to normal after treatment with cryptotanshinone (*p* < 0.01Figure 5(b)).

**Figure 6:**
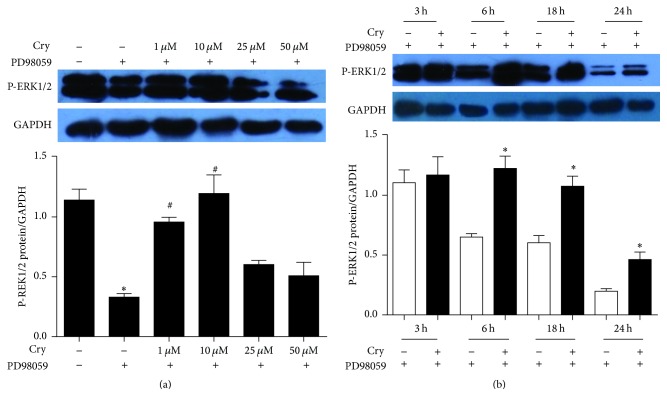
The effects on protein expression of P-ERK1/2 in pGCs treated with cryptotanshinone (values are mean ± SEM). (a) The protein level of P-ERK1/2 in pGCs when treated with 0 *μ*M, 1 *μ*M, 10 *μ*M, 25 *μ*M, and 50 *μ*M cryptotanshinone for 24 h. ^*∗*^*p* < 0.05 versus control I (Cry− and PD−); ^#^*p* < 0.05 versus control II (Cry− and PD+) (one-way ANOVA). (b) The protein level of P-ERK1/2 in androgen excess cells when treated with 10 *μ*M cryptotanshinone for 3 h, 6 h, 18 h, and 24 h. ^*∗*^*p* < 0.05 versus Cry− (one-way ANOVA). All experiments were repeated at least three times.

Interestingly, the P-ERK1/2 level decreased upon exposure to PD98059 (*p* < 0.05) (Figure 6(a)) and returned to normal after treatment with 1 *μ*M or 10 *μ*M cryptotanshinone (*p* < 0.05Figure 6(a)). The time course data showed that 10 *μ*M cryptotanshinone returned P-ERK1/2 levels to normal after 6 h and 18 h (*p* < 0.05; Figure 6(b)). Based on these results, we speculated that the ERK, c-Fos, and CYP17 proteins might play a role in androgen synthesis in response to PD98059 and that cryptotanshinone might reduce androgen levels by regulating the expression of these molecules.

### 3.4. The Potential Relationships among ERK, c-Fos, and CYP17

**Figure 7:**
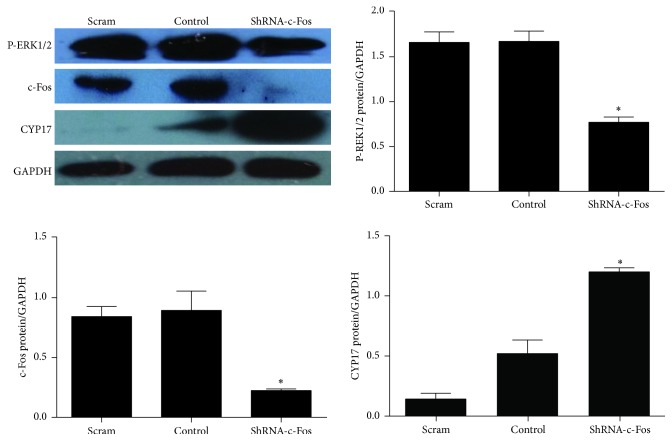
Protein expression of ERK, c-Fos, and CYP17 in pGCs after transfection with c-Fos shRNA for 72 h (values are mean ± SEM). ^*∗*^*p* < 0.05 versus control (one-way ANOVA). All experiments were repeated at least three times.

**Figure 8:**
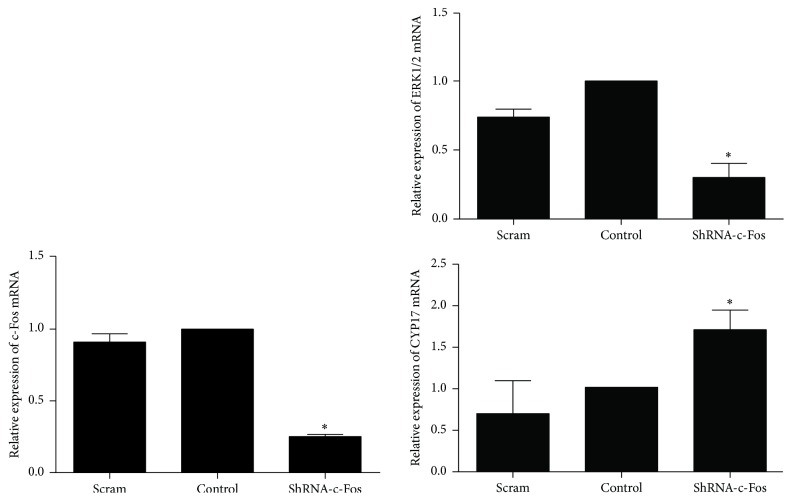
The mRNA expression of ERK, c-Fos, and CYP17 in pGCs after transfection with c-Fos shRNA for 72 h (values are mean ± SEM). ^*∗*^*p* < 0.05 versus control (one-way ANOVA). All experiments were repeated at least three times.

To determine whether ERK, c-Fos, and CYP17 interact in regulating androgen synthesis and to confirm the potential molecular mechanism behind decreased androgen synthesis by cryptotanshinone, we performed a c-Fos gene silencing experiment. After 72 hours of infection, the protein levels of P-ERK1/2 and c-Fos were significantly reduced in the shRNA–c-Fos group compared to the scrambled group (*p* < 0.01; Figure 7), and the protein level of CYP17 was significantly increased in the shRNA–c-Fos group compared to controls (*p* < 0.01; Figure 7). The same results were seen for mRNA expression of ERK1/2, c-Fos, and CYP17 (*p* < 0.05; Figure 8). These results implied that there is a negative correlation between c-Fos/ERK1/2 and CYP17 in pGCs and a positive correlation between c-Fos and ERK1/2.

## 4. Discussion

We have successfully isolated and identified pGCs and constructed an androgen excess cell model using the mitogen-activated protein kinase (MAPK) inhibitor PD98059 in pGCs. We used this hyperandrogen cell model to evaluate the effectiveness of reducing androgen excess with cryptotanshinone. Our results suggest that cryptotanshinone can reduce androgen levels and that the mechanism of the action might be through regulation of the expression of ERK/c-Fos/CYP17.

### 4.1. Cryptotanshinone Can Reverse the Androgen Excess Phenotype in pGCs Induced by PD98059

PD98059—a pharmacological inhibitor of MAPK that prevents the activation of ERK1/2 by regulating MEK1/2—has been widely used to examine the role of the ERK1/2 pathway in cellular signaling [28–30]. Some studies have confirmed that inhibition of the MEK/ERK signaling pathway by PD98059 is associated with increased steroid biosynthesis [31, 32] and results in an 11-fold increase in CYP17 mRNA expression and a 13-fold increase in androstenedione production in granulosa cells [33]. The same result was found in adrenal cells in which a reduction in P-ERK1/2 was associated with increased CYP17 gene expression [34]. Other experiments showed that MAPK inhibitors can also induce androgen excess [23, 24]. Thus we cultured pGCs with MAPK inhibitor PD98059 to induce the androgen excess phenotype. We showed that the testosterone level was significantly increased in the culture medium and that the protein level of CYP17 was also significantly increased in cells after exposure to PD98059, but the protein level of P-ERK1/2 was decreased after exposure to PD98059. These results are consistent with previous studies and imply that there is a connection between aberrant P-ERK1/2 and androgen excess in pGCs.

Cryptotanshinone, a major active ingredient of tanshinone, is commonly used for the treatment of acne in China due to its antiandrogenic activity [35, 36]. The therapeutic effect of tanshinone in acne treatment is based on a reduction in testosterone levels [19], and the therapeutic effect in the case of excessive ovarian androgens is also based on a reduction of testosterone levels [22]. In this study, we treated the androgen excess phenotype with cryptotanshinone to determine if cryptotanshinone could reduce the androgen level in pGCs, and our results confirmed that both the testosterone level and the protein level of CYP17 were decreased after treatment with cryptotanshinone. These results suggest that cryptotanshinone can effectively reduce androgen excess that originates in the ovary.

### 4.2. The Potential Molecular Mechanism of Decreasing Androgen Excess by Cryptotanshinone

The MAPK/ERK pathway is an important signaling cascade that controls cellular proliferation and differentiation. The phosphorylation and activation of MEK1/2 can result in activation of the ERK1/2 pathway [26] that then stimulates the expression and activity of a number of transcription factors, including members of the Jun and Fos families [37–40]. c-Fos is an integral activator protein transcription factor, and it has been shown that impaired c-Fos activity is involved in the pathogenesis of aberrant androgen excess conditions such as PCOS [41, 42]. The ERK pathway is one of the key regulators of c-Fos expression and function [43, 44]. One study demonstrated that inhibition of the ERK pathway resulted in diminished c-Fos levels and a concomitant increase in CYP17 in granulosa cells. This resulted in a subsequent increase in androstenedione synthesis in granulosa cells and suggested that the ERK pathway might be one mechanism responsible for the inhibition of CYP17 production in the granulosa cells and that c-Fos might be one of the factors responsible for CYP17 repression and, therefore, suppression of androgen production in granulosa cells [33]. This is consistent with the results of our study. We also demonstrated that inhibition of the ERK pathway resulted in significantly increased testosterone levels and CYP17 protein levels in pGCs (Figures 2 and 3) and significantly decreased protein levels of c-Fos and P-ERK1/2 (Figures 5(a) and 6(a)).

We treated the androgen-producing cells with cryptotanshinone to explore its effectiveness and potential molecular mechanism in reducing androgen excess. We showed that the protein levels of P-ERK1/2 and c-Fos were both significantly increased after treatment with cryptotanshinone (Figures 5 and 6) and that the protein level of CYP17 and the synthesis of testosterone were both significantly decreased (Figure 4). Thus we have confirmed that cryptotanshinone can significantly reduce androgen levels. We speculated that the potential molecular mechanism might be through upregulation of phosphorylation of ERK1/2 that leads to upregulation of c-Fos protein expression and, finally, to the downregulation of CYP17 protein expression that results in decreased androgen levels. To further confirm this hypothesis, we silenced the c-Fos gene in pGCs and found that both the CYP17 protein and the CYP17 mRNA levels were significantly increased and that the P-ERK1/2 protein level and the ERK1/2 mRNA level were significantly decreased (Figures 7 and 8). These results implied that there is a negative correlation between c-Fos/ERK1/2 and CYP17 in pGCs and a positive correlation between c-Fos and ERK1/2. All of these results suggest that PD98059 can induce androgen excess by inhibiting ERK1/2 phosphorylation, which leads to reduced c-Fos protein expression and subsequent upregulation of CYP17 protein expression. Cryptotanshinone, then, reduces androgen excess by increasing the phosphorylation of ERK1/2 that in turn promotes the expression of c-Fos protein and leads to reduced protein expression of CYP17.

Other studies have obtained the same results as ours. They have shown that ERK1/2 phosphorylation is decreased in PCOS theca cells compared to normal theca cells and that CYP17 mRNA and androgen synthesis are increased in PCOS theca cells. CYP17 production and androgen synthesis could be inhibited by c-Fos in PCOS theca cells [41]. The same result was confirmed in granulosa cells in which CYP17 production was also suppressed by c-Fos [33]. Under normal conditions, granulosa cells exhibit significantly higher steady-state levels of c-Fos than theca cells and do not secrete androgens [41]. However, microarray analysis demonstrates that the level of c-Fos transcription is decreased in PCOS ovaries compared to normal ovaries, especially in the granulosa cell layer, and that this is a contributing factor to the hyperandrogenism in PCOS [45].

Based on previous studies and our study, we suggest that cryptotanshinone could be used to reduce the androgen excess of PCOS and that the mechanism of reducing the androgen excess with cryptotanshinone in PCOS patients possibly functions through increasing the expression of P-ERK1/2 and promoting the expression of c-Fos that then reduces the expression of CYP17 and results in decreased androgen levels in PCOS granulosa cells.

## 5. Conclusion

In summary, our results demonstrate that cryptotanshinone can reduce androgen excess in pGCs. The possible mechanism of action is through upregulating the expression of P-ERK1/2 and c-Fos protein levels that leads to decreased CYP17 protein level and decreased synthesis of androgen. Our study suggests that cryptotanshinone might be a novel method to treat the androgen excess in PCOS patients, but our results in pGCs need to be further demonstrated in granulosa cell of PCOS patients.
